# Herbicide Persistence in Seawater Simulation Experiments

**DOI:** 10.1371/journal.pone.0136391

**Published:** 2015-08-27

**Authors:** Philip Mercurio, Jochen F. Mueller, Geoff Eaglesham, Florita Flores, Andrew P. Negri

**Affiliations:** 1 Australian Institute of Marine Science, Townsville, Queensland, Australia; 2 The University of Queensland, National Research Centre for Environmental Toxicology, Coopers Plains, Queensland, Australia; Northwest Fisheries Science Center, NOAA Fisheries, UNITED STATES

## Abstract

Herbicides are detected year-round in marine waters, including those of the World Heritage listed Great Barrier Reef (GBR). The few previous studies that have investigated herbicide persistence in seawater generally reported half-lives in the order of months, and several studies were too short to detect significant degradation. Here we investigated the persistence of eight herbicides commonly detected in the GBR or its catchments in standard OECD simulation flask experiments, but with the aim to mimic natural conditions similar to those found on the GBR (i.e., relatively low herbicide concentrations, typical temperatures, light and microbial communities). Very little degradation was recorded over the standard 60 d period (Experiment 1) so a second experiment was extended to 365 d. Half-lives of PSII herbicides ametryn, atrazine, diuron, hexazinone and tebuthiuron were consistently greater than a year, indicating high persistence. The detection of atrazine and diuron metabolites and longer persistence in mercuric chloride-treated seawater confirmed that biodegradation contributed to the breakdown of herbicides. The shortest half-life recorded was 88 d for growth-regulating herbicide 2,4-D at 31°C in the dark, while the fatty acid-inhibitor metolachlor exhibited a minimum half-life of 281 d. The presence of moderate light and elevated temperatures affected the persistence of most of the herbicides; however, the scale and direction of the differences were not predictable and were likely due to changes in microbial community composition. The persistence estimates here represent some of the first appropriate data for application in risk assessments for herbicide exposure in tropical marine systems. The long persistence of herbicides identified in the present study helps explain detection of herbicides in nearshore waters of the GBR year round. Little degradation of these herbicides would be expected during the wet season with runoff and associated flood plumes transporting a high proportion of the original herbicide from rivers into the GBR lagoon.

## Introduction

### Runoff affecting the Great Barrier Reef

The Great Barrier Reef World Heritage Area (GBRWHA) consists of 3000 reefs spanning 1800 km along the Queensland coast. Extensive agriculture including sugarcane, bananas, horticulture, grazing, and plantation forestry have been developed on cleared land in catchments that drain into the GBR lagoon and herbicides are applied to control weeds and grasses on this land [1, 2]. The main transport mechanism for offsite migration of herbicides is via intense monsoonal rainfall events during the summer wet season [3]. River runoff then delivers excess sediments, nutrients and pesticides which contribute to reduced water quality in nearshore and reefal environments [1, 4–6]. The transport of contaminants into the GBR lagoon can be extensive with flood plumes migrating 40–50 km to the mid-shelf regions of the GBR [7, 8], and sometimes as far as 130 km offshore to the outer GBR [6]. River plumes from multiple catchments can also combine, forming cumulative events along 1200 km of the GBR lagoon [6–9].

### PSII herbicides in the GBR

While a wide spectrum of pesticides have been detected in waters of the GBR and its catchments, herbicides are often more mobile than most insecticides and are consequently more often detected in the river mouths and the ocean [2, 4, 9, 10]. The most commonly detected herbicides in the GBR act through blocking electron transport in photosynthesis and specifically within photosystem II (PSII) [11]. An estimated 30,000 kg yr^-1^ of PSII herbicides (atrazine, diuron, hexazinone, tebuthiuron, simazine and ametryn) are transported into to the GBR lagoon annually [5, 12, 13]. The Mackay-Whitsundays (10,000 kg yr^-1^) and Wet Tropics (12,000 kg yr^-1^) regions account for the majority of the load to the GBR lagoon [12]. Individual PSII herbicides have been detected in in nearshore envrionments at concentrations exceeding 0.9 μg l^-1^, the current guidleline intended to protect 99% of marine species [2, 14–16]. Other non-PSII herbicides such as metolaclor, 2,4-D and glyphosate are also frequently detected across the GBR catchments [2]. The detection of PSII herbicides, far from source rivers in the GBR and during the dry season, many months after the last rainfall and flood plume events [1, 4, 9, 14, 17] indicates that many of these compounds may be relatively persistent in tropical marine waters.

### Herbicide persistence in seawater

Persistence of chemicals is generally measured using flask experiments under controlled conditions, which can provide consistent and repeatable conditions. Despite their common use for over five decades, we found only seven studies that measured and reported half-lives of PSII herbicides atrazine, simazine, diuron and the non-PSII herbicides 2,4-D and glyphosate in seawater (Table 1). Experimental parameters including initial herbicide concentration, temperature, light/UV, natural versus filtered seawater, and duration varied considerably between these studies and could affect reported persistence. For example, herbicide concentrations in the marine environment are generally less than 10 μg l^-1^ [4, 13]; however, most previous studies tested degradation of far higher concentrations (Table 1). Very high starting concentrations can potentially result in: (i) the development of unrealistic capacity in microbial communities to metabolise this dominant carbon source, (ii) increased lag times in degradation as microbial communities struggle to adapt to high herbicide concentrations, or (iii) toxicity towards some components of the native microbial communities [18]. The majority of previous herbicide degradation experiments in seawater were performed under temperate conditions 15°C–20°C (Table 1). In contrast, seawater temperatures during peak herbicide contamination periods in tropical systems are generally between 24°C–31°C [19] and higher metabolic rates in microbial communities under these conditions may contribute to lower persistence under these warmer conditions [20]. The presence of light can also have an impact on persistence due to contributions from photodegradation or changes in microbial communities. For example, Navarro et al. [21] demonstrated almost 3-fold more rapid degradation of high atrazine concentrations under full sunlight conditions (Table 1). Herbicides transported into the GBR via highly turbid flood plumes are exposed to low-moderate levels of light and UV penetration is likely to be highly attenuated under these conditions [22]. Some of the previous persistence experiments were conducted for short durations of 20 days for atrazine and simazine [23] and 42 days for diuron [24], not long enough to detect significant degradation. Only longer experimental durations of more than 120 days yielded half-life estimates for atrazine and simazine [21]. While many conditions listed in Table 1 may not have been ideal to estimate herbicide persistence in tropical waters, most were conducted using unfiltered or partially-filtered seawater which is suitable to test the ability of natural microbial communities to degrade herbicides (OECD, 2005).

**Table 1:** Published literature on the degradation half-life experiments in seawater.

Herbicide	Half-life (days)	Initial conc.(μg l^-1^)	Light	Temperature	Water filtration	Reference
**Diuron**	No degradation for 42 d	1000	Light	15°C	unfiltered	[24]
**Atrazine**	SL = 79	5000	SL SD	20°C	unfiltered	[21]
	SD = 206					
**Atrazine**	S = 79	5000	Light	S = 20°C	unfiltered	[20]
	ST = 65			ST = 40°C		
**Atrazine**	No degradation for 20 d	20	Light	20°C	filtered 0.45μm and unfiltered	[23]
**Atrazine**	S = 56	~8500	Light	22°C	unfiltered	[36]
**Simazine**	SL = 29	5000	SL SD	20°C	unfiltered	[21]
	SD = 49					
**Simazine**	S = 29	5000	Light	S = 20°C	unfiltered	[20]
	ST = 28			ST = 40°C		
**Simazine**	No degradation for 20 d	20	Light	20°C	filtered 0.45um and unfiltered	[23]
**2,4-D**	SL = 11.1	2000	SLSD	20°C	unfiltered	[37]
	SD = 31.2					
**Glyphosate**	D25 = 267	10	Dark	25°C	20 μm filtered	[29]
	D31 = 315		Dark	31°C		
	L25 = 47		Light	25°C		

SL = seawater light, SD = seawater dark, S = seawater, ST = seawater at elevated temperature.

Assessing the risks posed by the range of herbicides commonly detected in the GBR lagoon is difficult considering the absence of appropriate persistence data (see above). While there is a far greater body of studies that have investigated herbicide persistence in freshwater and soil systems [25], there is a requirement for data relevant to tropical marine systems. The aim of this study is to determine persistence, including factors that affect persistence, of herbicides in tropical marine water and provide long needed information towards understanding the fate of herbicides when they enter the GBR.

## Materials and Methods

The experimental approach that was taken follows the standard OECD [26] guidelines for testing biodegradation in seawater modified to OECD [27] “simulation test” criteria which utilise environmentally relevant conditions rather than those conducive to bioremediation. The experiments were conducted in coastal seawater containing native bacterial communities without addition of nutrients or artificial inoculum to best mimic ecological conditions and used low initial concentrations (4–18 μg l^-1^), relevant to the receiving waters in the field. Background (control) solutions were analysed for herbicides and contained < 0.1 μg l^-1^ (below analytical detection limit) and did not contribute to the original concentrations.

### Experiment 1

Nearshore coastal seawater was collected at the Australian Institute of Marine Science (19°16’ S, 147° 03’ E), Cape Cleveland, QLD under the permit G12/35236.1 issued by the Great Barrier Reef Marine Park Authority. The seawater was filtered to 0.45 μm to remove all particulates and added to individual 500 ml Erlenmeyer flasks (300 ml final volume). The sample treatments were spiked to a final concentration of ~10 μg l^-1^ for each herbicide (Table 2) and the flasks stoppered with autoclaved cotton bungs to allow for aerobic conditions. Herbicide standards (98.5–99.9%) were purchased from Sigma-Aldrich, added to 2 ml of the carrier solvent ethanol (to assist in solubility), and made to 5 mg l^-1^ concentration with Milli-Q water. The same volume of ethanol (final less than 0.03% v/v) was added to all flasks, including controls for consistency between treatments. Triplicate flasks were shaken at 25°C and 100 rpm in the dark using an Innova 44, incubator shaker. One series of flasks contained a mixture of the six PSII herbicides (ametryn, atrazine, diuron, hexazinone, simazine, tebuthiuron) and the second series of flasks the same herbicide mixture with the addition of 45 mg l^-1^ mercuric chloride (MC) to eliminate microbial activity (Table 2) [28]. Sample treatment flasks were weighed before sampling to monitor evaporation losses for concentration adjustments. Flasks were topped up with fresh sterile water (Milli-Q) and any losses were compensated for during calculations. Experiment 1 (pilot) examined the degradation of six PSII herbicides over 60 d (Table 2). The 60 day experiment length was set as the maximum by following the OECD method. The purpose of this experiment was to test whether bacteria contributed to biodegradation of these herbicides. Microbial activity is eliminated in the presence of MC and to inform the second experiment which was to be conducted over a longer period.

**Table 2:** Experimental details of two degradation experiments investigating herbicide degradation in (1) filtered seawater and (2) unfiltered seawater.

Experiment	Experiment 1 (Pilot)	Experiment 2
**Experimental units**	3 replicate flasks	3 replicate flasks
**Herbicides**	PSII mix (ametryn, atrazine, diuron, hexazinone, simazine, tebuthiuron)	
		Atrazine
		Diuron
	PSII mix (as above) with MC	Hexazinone
		Tebuthiuron
	Control (no herbicides)	Metolachlor
		2,4-D
		Control (no herbicides)
**Coastal seawater**	0.45 μm filtered	20 μm filtered
**Light conditions**	Dark only	Dark or 40 μE light
**Temperature**	25°C	25°C or 31°C
**Duration**	60 days	365 days
**Days sampled**	0,7, 28, 60	0, 28, 60, 120, 180, 240, 300, 365

All flasks were shaken at 100 rpm. MC = mercuric chloride.

### Experiment 2

The methodology for this second experiment was modified slightly from Experiment 1 (pilot). Coastal seawater was collected and filtered to 20 μm to introduce the total bacterial diversity from this environment. The filtered seawater also included other active substances such as fine mineral/organic particles (<20 μm) and dissolved organic carbon; however, we expect minimal adsorption to particles since the herbicides are characterized by low partition coefficients (log K_OW_ < 3).

The 20 μm-filtered seawater (300 ml in three replicate 500 ml Erlenmeyer flasks) was capped with autoclaved and oven dried silicone bungs. A total of seven herbicides were included in this experiment, each with an initial concentration of 4–18 μg l^-1^. Incubator conditions and results for glyphosate were published previously in Mercurio et al. [29]. In brief, the degradation study was conducted under three test conditions (Table 2) in three separate temperature-regulated incubator shakers (set to 25°C or 31°C). An even light environment of 40 μmol photons m^-2^s^-1^ over a 12:12 light day cycle was provided in one 25°C shaker using cool white LED strips. This was equivalent to 1.7 mol photons m^-2^day^-1^, comparable to shallow 3–6 m depths on turbid nearshore reefs of the GBR during the wet season [30]. The experiment flasks were randomised after every sampling period. Seawater was sampled 19 times over the year-long study, including the 60 day time point to allow for comparisons with Experiment 1. Experiment 2 examined the year-long degradation of four PSII herbicides along with 2,4-D and metolachlor in 20 μm filtered coastal water under three different light and temperature scenarios: (1) in the dark at 25°C which corresponds to the mean annual seawater temperature on the GBR [19]; (2) in low light conditions at 25°C and (3) in the dark and at 31°C which is a summer maximum temperature for nearshore areas of the mid-northern regions of the GBR [19] (Table 2).

### Herbicide analysis

Aliquots (2 ml) were transferred into 4 ml amber glass vials and spiked with a surrogate standard, d5-Atrazine (10 μg l^-1^). The final concentration of the surrogate standard was 5 μg l^-1^. The samples were stored at -20°C and filtered prior to analysis. After each experiment had ended, samples were run in sequential order. The herbicide and degradation product concentrations were determined by HPLC-MS/MS using an AB/Sciex API5500Q mass spectrometer (AB/Sciex, Concord, Ontario, Canada) equipped with an electrospray (TurboV) interface and coupled to a Shimadzu Prominence HPLC system (Shimadzu Corp., Kyoto, Japan). Column conditions were as follows: Phenomenex Synergi Fusion RP column (Phenomenex, Torrance, CA) 4 μm 50 x 2.0 mm, 45°C, with a flow rate of 0.4 ml min^-1^. The column was conditioned prior to use and for analyte separation required a linear gradient starting at 8% B for 0.5 min, ramped to 100% B in 8 min then held at 100% for 2.0 min followed by equilibration at 8% B for 2.5 min (A = 1% methanol in HPLC grade water, B = 95% methanol in HPLC grade water, both containing 0.1% acetic acid). The mass spectrometer was operated in the positive ion, multiple reaction-monitoring mode (MRM) using nitrogen as the collision gas. Analyte quantification and confirmation ions are listed in Supporting Information S1 Table.

Positive samples were confirmed by retention time and by comparing transition intensity ratios between the sample and an appropriate concentration standard from the same run. Sample were reported as positive if the two transitions were present, retention time was within 0.15 minutes of the standard and the relative intensity of the confirmation transition was within 20% of the expected value. The value reported was that for the quantitation transition. The limit of detection for this method was typically less than 0.1 μg l^-1^, yielding a reporting limit of 0.2 μg l^-1^. Response was linear to at least 20 μg l^-1^. Sample sequences were run with a standard calibration at beginning and end of sequence with additional mid-range standards run every 10 samples.

### Water chemistry

The physical/chemical characteristics of the filtered seawater used in the experiment were measured, including dissolved oxygen, salinity and pH. Dissolved oxygen and pH were measured before and at the end of each experiment. The salinity remained between 33 and 34 PSU throughout and the pH and dissolved oxygen (DO) levels of seawater in the flasks were similar between controls, treatments and freshly-collected natural seawater at the end of the 60 and 365 day experiments (in S2 and S3 Tables). Water chemistry was analysed using standard techniques and is reported in S3 Table.

### Flow cytometry

Flow cytometry was used to quantify the microbial populations in the seawater used in the experiment. Samples were fixed with 5% formaldehyde and stored at 4°C. Sub-samples were stained using Sybr Green, diluted to 1:10,000, and allowed to develop in the dark for 30 min. Samples were run using a BD Accuri C6 cytometer (BD Biosciences, CA, USA) equipped with a red and blue laser (488 nm, 50 mW maximum solid state; 640 nm, 30 mW diode) and standard filter setup. Flow rate was 14 μl min^−1^, 10-μm core. The natural microbial community populations and their abundances were measured for the initial seawater as well as treatments for the experiment using the Accuri CFlow plus software. On the basis of the flow cytometry results, the seawater in flasks contained similar bacterial abundance at the end of the experiment compared with natural seawater. The values measured were consistent with the range expected for seawater (S2 Table) [31–33].

### Data analysis

Half-life (*t*_1/2_) calculations assumed first order kinetics and were estimated from the decline in experiment concentration of herbicide in seawater using the rate constant (*k*) slope of the data obtained from plots of the natural logarithm of the concentrations versus time (*t*), where *t*_1/2_ = ln(2)/*k* [34, 35]. First order kinetics were applied as outlined in standard OECD protocols [27, 28], and previous degradation experiments on herbicides [20, 36–38]; however, zero order kinetics may apply for low concentrations of a pesticide in the presence of other natural carbon sources [39] so this approach was also tested. Half-lives for zero order kinetics were obtained by plotting the concentration vs time: *t*_1/2_ = 0.5 *C*_*0*_/*k*_0_, where *C*_*0*_ is the initial concentration and *k*_*0*_ is the slope. Comparisons between approaches can be found in S4 Table (Experiment 1) and S5 Table (Experiment 2), revealing good fits for both zero- and first-order models (similar r^2^ values for linear plots). Both models predicted similarly long half-lives (on average 25% and 9% difference between approaches in Experiment 1 and Experiment 2 respectively) and we have subsequently presented data from the more conventional first-order approach. Herbicide concentrations below the reporting limit were removed from the analysis. The concentration data was analysed by repeated measures analysis of variance (ANOVA) using Number Cruncher Statistical System (NCSS 9) (Statistical and Power Analysis Software) across the time points sampled. Significance was determined if the resulting p-value was <0.05 between the initial concentration at day 0 and specified time point (e.g. day 60, day 365). The probability that *t*_1/2_ was statistically different between light and temperature treatments were tested by comparing the heterogeneity of the regression (whether the slopes were different) using a two-tailed test [40] in Graph Pad Prism V 6.0. When p < 0.05 the slopes were considered significantly different.

## Results

### Experiment 1

**Fig 1:**
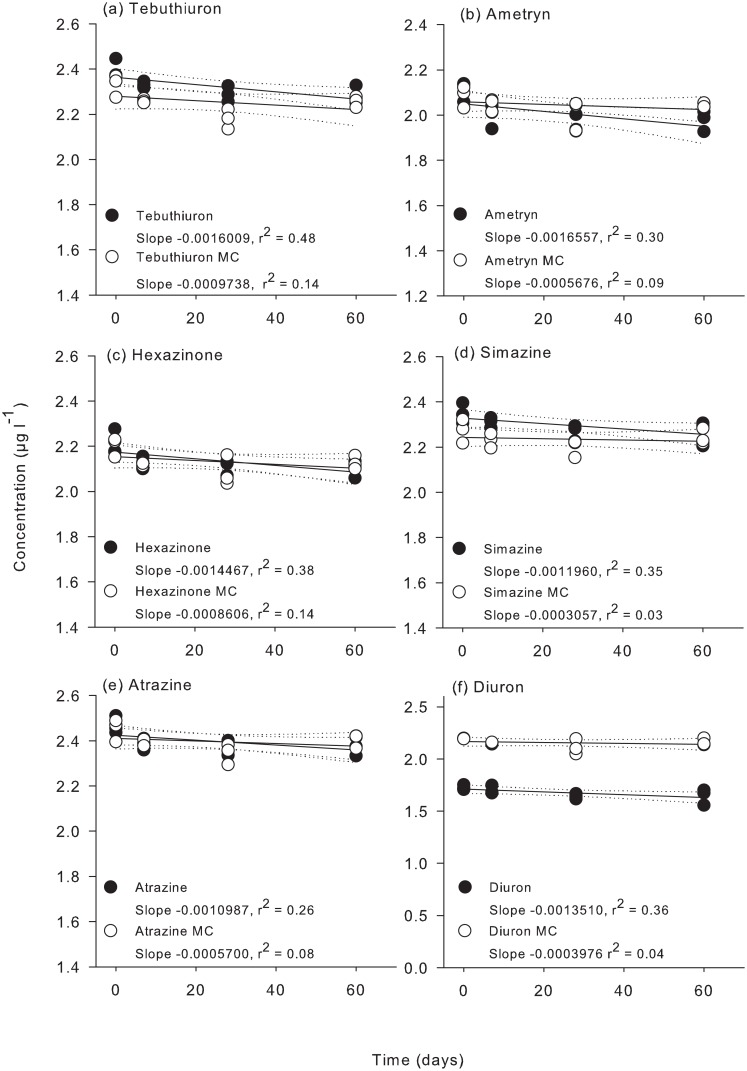
Experiment 1 half-life results. ln(x) concentration of the herbicide mixture: (a) tebuthiuron, (b) ametryn, (c) hexazinone, (d) simazine, (e) atrazine, and (f) diuron over 60 days. SE = Standard error. MC = mercuric chloride treatment.

Significant decreases in all PSII herbicide concentrations over the 60 d duration were recorded in the presence of bacteria with the exception of diuron (Fig 1, S6 Table). The decrease in herbicide concentrations in the presence of bacteria resulted in half life estimates between 420 for ametryn and 631 d for atrazine (Table 3). Repeated measures ANOVA indicated that there was no significant degradation of any of the PSII herbicides in the presence of mercuric chloride over 60 d (Fig 1, S6 Table). Metabolites of diuron (3,4 dichloroaniline) and atrazine (desethyl atrazine and desisopropyl atrazine) were not observed in either treatment.

**Table 3:** Experiment 1 and 2 half-lives.

	Expt. 1, 60 d		Expt. 2, 365 d		
Herbicide	Dark 25°C	Dark 25°C + MC	Dark, 25°C	Light, 25°C	Dark, 31°C
**Diuron**	NS	NS	1568 ± 222^a^	556 ± 14^b^	818 ± 51^b^
**Atrazine**	631 ± 491	NS	1606 ± 129^a^	2089 ± 338^a,b^	2066 ± 154^b^
**Hexazinone**	479 ± 240	NS	2792 ± 172^a^	2799 ± 467^a^	1434 ± 114^b^
**Tebuthiuron**	433 ± 150	NS	5214 ± 705^a^	2650 ± 291^b^	2840 ± 358^b^
**Ametryn**	419 ± 264	NS	-	-	-
**Simazine**	579 ± 294	NS	-	-	-
**Metolachlor**	-	-	281 ± 1^a^	320 ± 61^a^	298 ± 12^a^
**2,4-D**	-	-	146 ± 19^a^	494 ± 72^b^	88 ± 6^a^

D25 = 25°C, dark; D31 = 31°C, dark; L25 = 25°C. MC = mercuric chloride. SE = Standard Error. Significant degradation after 60 and 360 d when p < 0.05 (repeated measures ANOVA, S6 Table), NS = not significant.–indicates herbicide not tested. The superscripts a,b represent significantly different slopes in Figs 3–5 graphs (S7 Table), indicating differences in persistence between treatments for that herbicide.

### Experiment 2

**Fig 2:**
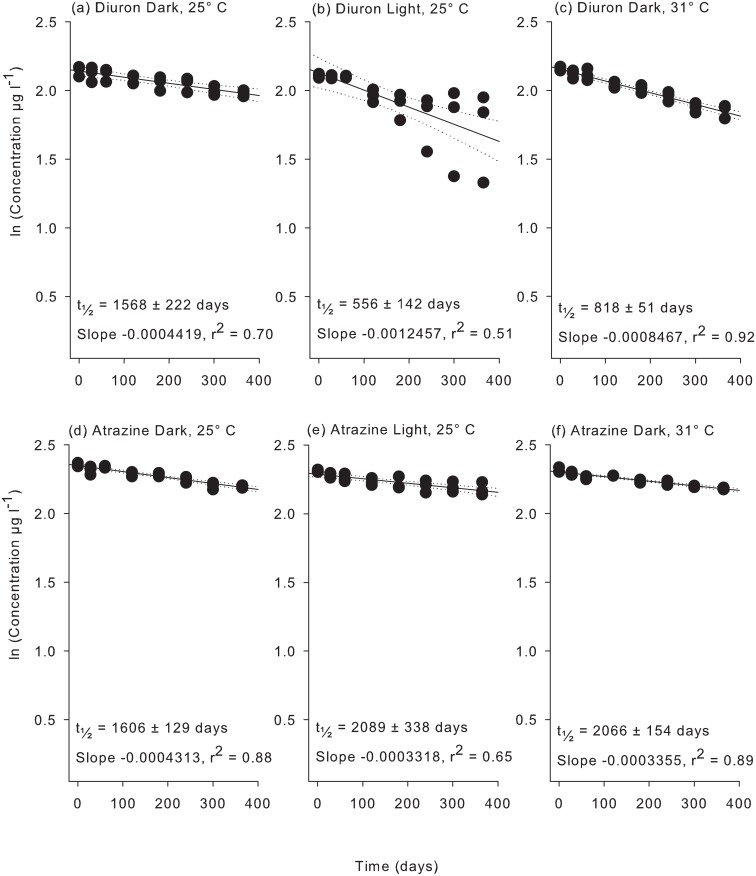
Experiment 2 half-life results for diuron and atrazine. ln(x) concentration of individual herbicide treatments: (a) diuron dark 25°C, (b) diuron light 25°C, (c) diuron dark 31°C, (d) atrazine dark 25°C, (e) atrazine light 25°C, and (f) atrazine dark 31°C over 365 days. Dashed lines represent 95% confidence intervals. Half-life reported ± SE.

**Fig 3:**
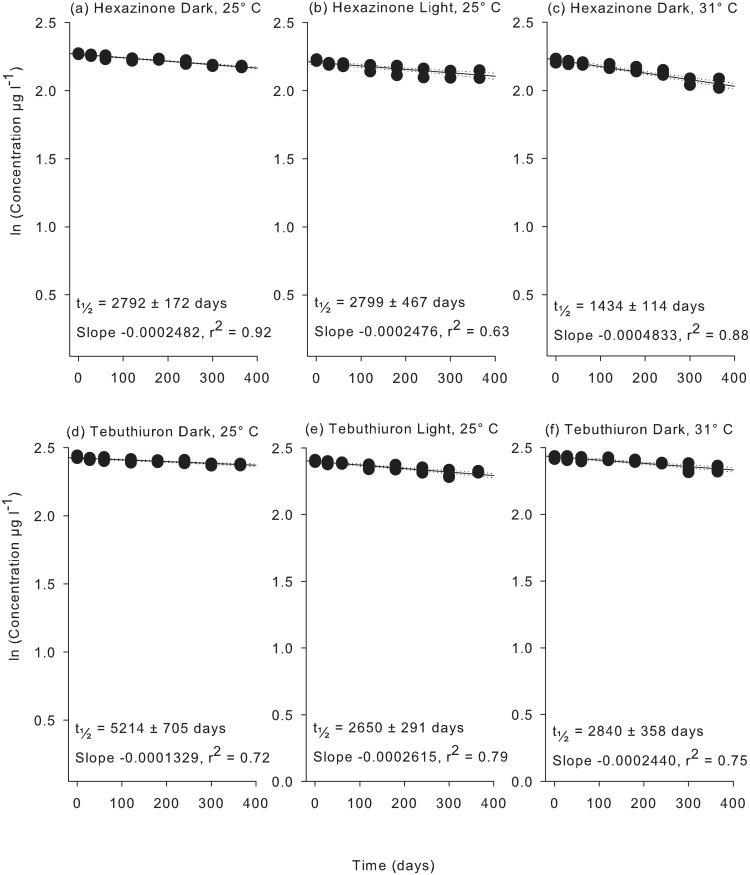
Experiment 2 half-life results for hexazinone and tebuthiuron. ln(x) concentration of individual herbicide treatments: (a) hexazinone dark 25°C, (b) hexazinone light 25°C, (c) hexazinone dark 31°C, (d) tebuthiuron dark 25°C, (e) tebuthiuron light 25°C, and (f) tebuthiuron dark 31°C over 365 days. Dashed lines represent 95% confidence intervals. Half-life reported ± SE.

**Fig 4:**
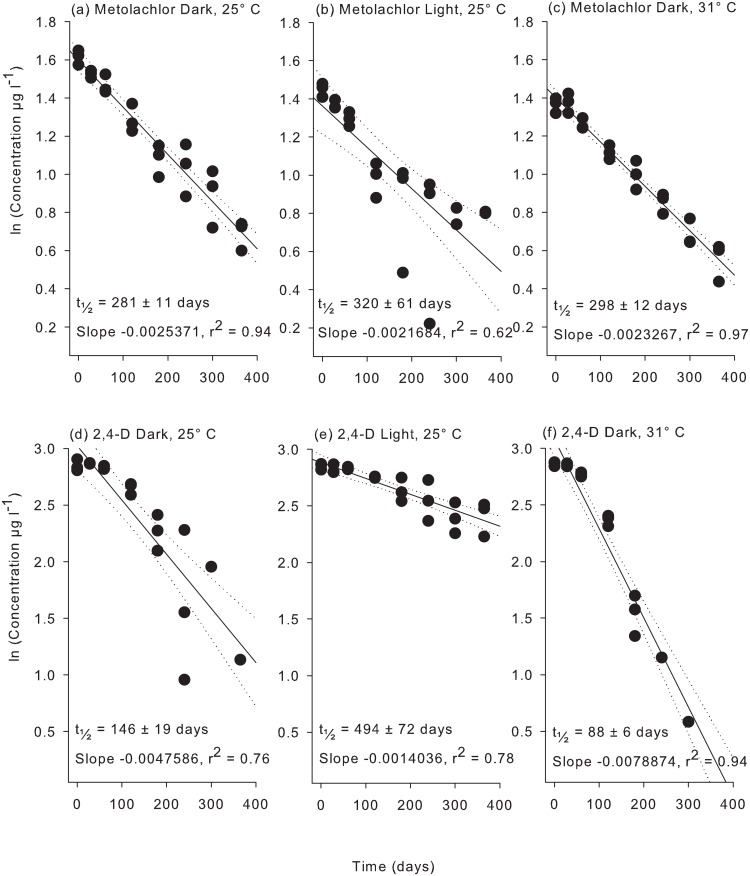
Experiment 2 half-life results for metolachlor and 2,4-D. ln(x) concentration of individual herbicide treatments: (a) metolachlor dark 25°C, (b) metolachlor light 25°C, (c) metolachlor dark 31°C, (d) 2,4-D dark 25°C, (e) 2,4-D light 25°C, and (f) 2,4-D dark 31°C over 365 days. Dashed lines represent 95% confidence intervals. Half-life reported ± SE.

Very little degradation of the PSII herbicides was observed (< 6%) over the first 60 d of the second flask experiment using 20 μm-filtered coastal water and less than 15% degradation of 2,4-D and metolachlor was evident over this period (Figs 2–4). After 365 d significant degradation was observed for all herbicides in coastal seawater (S6 Table). Diuron exhibited the shortest (t_½_ = 1568 d) and tebuthiuron the longest (t_½_ = 5214 d) half-lives of the PSII herbicide at 25°C under dark conditions (Table 3). The non-PSII herbicides metolachlor and 2,4-D degraded much more rapidly exhibiting half-lives of 281 d and 146 d respectively (Table 3).

Exposure to light and temperature resulted in a significant difference in slopes, ln [concentration] versus time, for of all herbicides apart from metolachlor (S7 Table) but there was no clear pattern between persistence and temperature or light (Table 3). For example the most rapid degradation for diuron was in the light at 25°C (t_½_ = 556 d) whereas atrazine degraded most rapidly at 25°C in the dark (t_½_ = 1606 d). The ratios of half-lives between experimental conditions represented in Table 4 reveals that the effect of light and temperature is often around 2-fold (diuron, hexazinone and tebuthiuron) or greater (2,4-D).

**Table 4:** The ratio of half-lives between each treatment from experiment 2.

	Diuron	Atrazine	Hexazinone	Tebuthiuron	Metolachlor	2,4-D
**D25**	1.0^a^	1.0^a^	1.0^a^	1.0^a^	1.0^a^	1.0^a^
**L25**	0.4^b^	1.3^a,b^	1.0^a^	0.5^b^	1.1^a^	3.4^b^
**D31**	0.5^b^	1.3^b^	0.5^b^	0.5^b^	1.1^a^	0.6^a^

**Fig 5:**
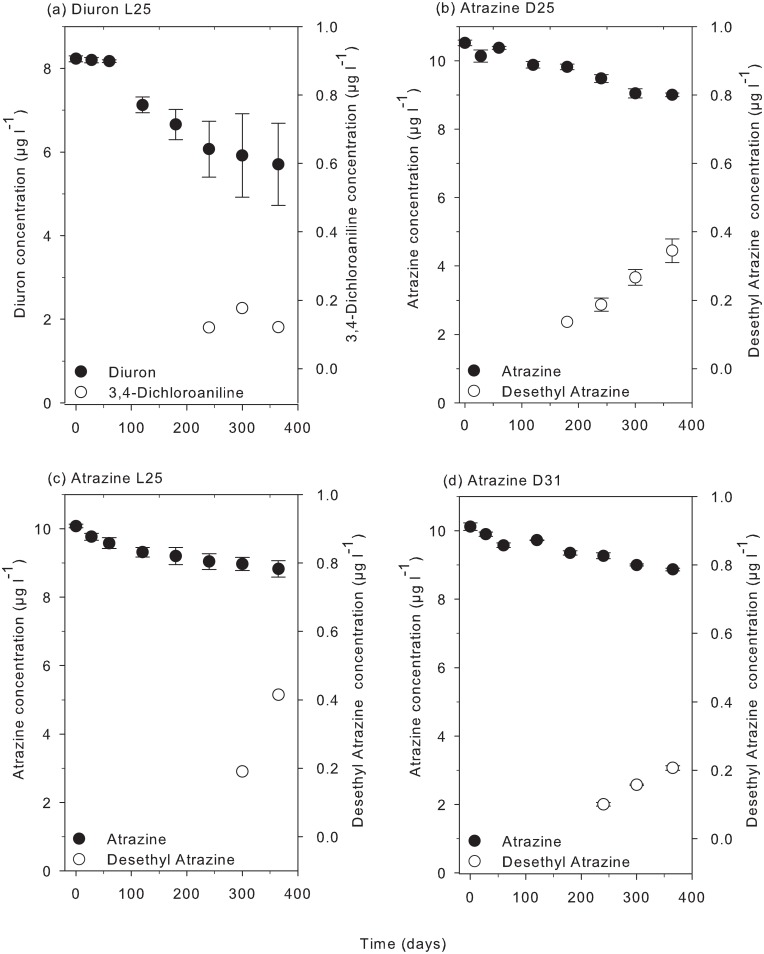
Concentration of metabolites of diuron and atrazine. Concentrations of individual herbicides including measured metabolites (a) diuron light 25°C, (b) atrazine dark 25°C, (c) atrazine light 25°C, and (d) atrazine dark 31°C, over 365 days. Bars represent ± SE. Note the concentration scales for the parent and metabolite are on opposite sides of each graph.

The superscripts a,b represent significantly different slopes (S7 Table) in Figs 3–5 graphs that in turn indicate differences in persistence between treatments for that herbicide.

Metabolites of diuron (3,4 dichloroaniline) and atrazine (desethyl atrazine) were observed in some of the treatments, confirming biodegradation of these herbicides. In the light treatment at 25°C diuron degraded into 3,4 dichloroaniline which reached a maximum concentration at 300 d (0.18 μg l^-1^, 2% of the applied parent) (Fig 5). Desethyl atrazine was detected under all three temperature-light conditions, reaching a maximum concentration of 0.42 μg l^-1^ (4% of the applied parent) at 365 d in the light treatment. Desisopropyl atrazine was not detected in any treatment.

## Discussion

Experiment 1 on the persistence of PSII herbicides in filtered seawater and in the presence and absence of microorganisms over 60 d revealed slow degradation by the native microbial community (half-lives of 419–631 d). The absence of significant degradation in the mercuric chloride-treated flasks indicated that hydrolysis of the PSII herbicides in seawater was negligible. In other studies and over shorter experimental durations, no degradation of diuron or atrazine was observed in seawater (Table 1) [23, 24]. However, experiments over 127 d have been used to determine the half-lives of atrazine and simazine (Table 1) [20, 21]. Degradation experiments that focus on remediation or identifying breakdown products may be brief due to the addition of nutrients and sewage-enriched microbial inoculum [38]. However, under less artificial conditions here the bacterial degradation of herbicides over a 60 d period was lower than 15% in all cases, indicating longer experiments are necessary to accurately estimate half-lives of these herbicides in marine environments.

Experiment 2, performed over a longer duration and using less filtered coastal waters, confirmed the high persistence of four PSII herbicides in the dark at 25°C (1568–5214 d) and a more rapid degradation of the non-PSII herbicides metolachlor and 2,4-D (146–281 d) (Table 3). These results indicate that, while the coastal GBR seawater contains high bacterial densities (> 2 x 10^6^ cells ml^-1^, S2 Table) that either (i) few bacteria are capable of metabolising the herbicides or (ii) other sources of carbon are preferentially utilised (and cycled) in the flasks. This experiment used natural coastal seawater that was only coarsely filtered to retain its native microbial community and excluded larger organisms such as zooplankton (> 20 μm) which may introduce large organic carbon loads and inconsistencies between flasks [27]. Without performing an exhaustive independent study of the microbial communities in the seawater we cannot be certain that known herbicide degraders were present; however, coastal GBR seawater usually has wide bacterial diversity between 500 to 7000 operational taxonomic units per litre [41, 42].

Herbicide concentrations used here (~ 10 μg l^-1^) were within the range of receiving waters that flow into the coastal GBR environment [2, 4, 13] and the LC-MS was sensitive enough to allow direct analysis of these environmental concentrations without pre-concentrations steps which can introduce further uncertainties. Differences in degradation rates between replicate flasks were potentially due to different microbial communities and unequal distribution of particulates and organic matter between flasks as the water was filtered to 20 μm and not likely to be absolutely uniform between replicates. Most other herbicide degradation experiments in seawater have used very high starting concentrations ≥ 5000 to μg l^-1^ [20, 21, 36]. As natural microbial degradation relies on indigenous bacterial communities to adapt to using the contaminant as a carbon source [18], unrealistically high concentrations of herbicides in solution could artificially influence natural microbial communities and subsequently affect apparent persistence and may even be toxic to some components of the communities. Furthermore, the herbicide concentrations here represent less than 1% of the total organic carbon (the majority of which was dissolved) in the seawater used in Experiment 2 (S2 Table), so bacteria had access to a selection of more abundant and natural carbon sources (which is the case in the natural coastal environment). In combination, the low herbicide concentrations and the abundance of other carbon sources are likely to have limited adaptation rate of the native microbial community in flasks to metabolise the herbicides more rapidly [39].

The identification of the atrazine metabolite desethyl atrazine (DEA) in all three treatment conditions (Fig 5) confirms biodegradation of this herbicide [18, 43]. The diuron metabolite 3,4 dichloroaniline (DCA) was also detected in one treatment and, although sometimes attributed to photodegradation and hydrolysis, DCA is usually considered a product of biodegradation [38, 44]. The degradation metabolites of other herbicides in the study were either unknown, below detection limits, or not analysed. The appearance of metabolites was late in the experiment ≥ 180 d and the concentrations were less than the reporting limit of 0.2 μg l^-1^. While these metabolites are sometimes detected in river runoff and in the GBR lagoon [2, 10, 45], the low concentrations detected and/or absence in the present study is likely to reflect the slow degradation of parent compounds into very low concentrations of metabolites that also start degrading (but at concentrations too low to detect). Greater initial herbicide concentrations would be needed in order positively identify more metabolites and the inclusion of labelled compounds and the establishment of mass balances would also further the understanding herbicide fate in the marine environment.

Degradation of herbicides was significantly influenced by temperature and light for all herbicides except metolachlor (Table 3 and S6 Table). Although photodegradation can contribute to the breakdown of several of these herbicides [46–49], the turbid conditions during flood plumes that transport herbicides into the marine environment are likely to greatly attenuate both visible and UV to levels well below the very high intensities that have been applied in previous studies. As the lights used in the present study emitted no UV component, any difference in persistence between the light (L25) and dark (D25) treatment must be due to changes in bacterial communities. In the presence of light, the two substituted urea herbicides diuron and tebuthiuron degraded at least twice as rapidly (yet still the half-lives remained long at 556 d for diuron and 2650 d for tebuthiuron, Table 4). Light had no impact on the persistence of atrazine, hexazinone and metolachlor (Table 4). Other studies have reported contrary effects in that atrazine and simazine degrade more rapidly under illumination [20, 21]. Differences are likely due to the combination of distinctive microbial communities, different starting concentrations and possibly light intensities [20, 21]. Under identical conditions to those in the present study Mercurio et al. [29] demonstrated more rapid degradation of glyphosate in the presence of low light levels. In contrast to the only other study on the degradation of 2,4-D in seawater [37], this herbicide degraded over 3-fold more slowly in the light (Table 4). Again differences in the amount of starting material (Dabrowska et al. [37] used 2000 μg l^-1^) and microbial community composition and/or light intensity may be responsible.

In the high summer temperature of nearshore waters (31°C, D31) the half-lives of diuron, hexazinone, tebuthiuron and 2,4-D were all shorter by a factor of ~2 (ie 40–60%), whereas the half-life of atrazine was slightly but significantly longer (i.e. 30%) in comparison to treatments at the mean annual temperature (25°C, D25) (Tables 3 and 4). More rapid degradation at higher temperatures may reflect more rapid metabolism by the microbial community; however decreases observed for atrazine suggests an influence of temperature on microbial community structure [50]. Although the increase of 6°C increased the degradation of diuron, hexazinone, tebuthiuron and 2,4-D, the persistence of the PSII herbicides remained long (t_1/2_ > 800 d), while the shortest half-life observed in this experiment of 88 d was observed for 2,4-D under these warmer conditions (Table 3). In a previous study atrazine may have degraded slightly more rapidly at an elevated temperature of 40°C as compared to 20°C (but this was not tested statistically) [20]. Atrazine degraded 30% more slowly at 31°C in the present study, probably due to (untested) differences in microbial composition. As with changes in light, the degradation rate of metolachlor was not impacted by these temperature conditions (Table 4).

Degradation studies which simulate natural conditions, such as low herbicide concentrations, relevant temperatures and light, natural microbial communities and no additional nutrients, provide the most realistic flask conditions for estimating persistence [27, 39]. This simulation study of degradation of multiple herbicides provides the most representative information to date on persistence in seawater, while a far greater body of studies on herbicide degradation have been performed in freshwater (See S8 Table for a relevant summary). In the present study very little degradation was recorded in seawater over the standard 60 d period (Experiment 1) and short experiments such as this are insufficient to estimate herbicide persistence in natural seawater. Over 360 d, the presence of moderate light and elevated temperatures affected the persistence of most of the herbicides; however, the scale and direction of the differences were not predictable and were likely due to changes in microbial community composition. Further simulation experiments are needed to assess the wide variety of herbicides detected in marine systems [2, 10], the results of which can be utilised in risk assessments for herbicides in the marine environment. Future studies should also include open tank or pond systems that include relevant sediments and a wider variety of light and temperature conditions to better mimic conditions in the field.

The half-lives of the PSII herbicides were all greater than a year, indicating the potential for long persistence in the marine environment. Little degradation of these herbicides would be expected during the wet season with runoff and associated flood plumes transporting a high proportion of the original herbicides in rivers into the GBR lagoon. The long persistence of herbicides identified here also helps explain detection of herbicides in nearshore waters of the GBR year round [1, 51] and raises the possibility of accumulation of herbicides and metabolites in some locations. Chronic exposure of GBR and catchment biota to PSII herbicides (from microbial communities to macrophytes) remains largely unstudied [52] and should be a future focus for research and risk assessment.

## Supporting Information

S1 Table Quantification and confirmation ions used for herbicide analysis. Compound 1 (e.g. Desisopropyl Atrazine 1) transitions used for quantitation 174.1/103, other compound designated 2 (Desisopropyl Atrazine 2) is for confirmation, 174.1/68.
(DOCX)

S2 Table Mean ± SE for each treatment for pH, dissolved oxygen (DO), and total bacterial counts for Experiment 1 and 2. (DOCX)

S3 Table Physical and chemical information for the 0.45 μm and 20 μm filtered seawater used in experiment 1 and experiment 2 respectively. NA = not applicable for 0.45 μm filtered seawater.
(DOCX)

S4 Table Results of Experiment 1 including first order half-life estimates, slopes, r^2^, average initial concentration, average final concentration, total average degradation. Additional results for Experiment 1 for zero order half-life estimates, r^2^, and the % difference between first order and zero order half-life estimates.
(DOCX)

S5 Table Results of Experiment 2 including first order half-life estimates, slopes, r^2^, average initial concentration, average final concentration, total average degradation. Additional results for Experiment 2 for zero order half-life estimates, r^2^, and the % difference between first order and zero order half-life estimates.
(DOCX)

S6 Table Results of statistical testing of Experiment 1 and Experiment 2. Repeated measures ANOVA testing significance of degradation over time.
(DOCX)

S7 Table Results of statistical testing of Experiment 2. Two-tailed test for differences between slopes (k).
(DOCX)

S8 Table Relevant literature on the degradation half-life experiments in freshwater. (DOCX)
