# Supplementary material for: Herbicide Persistence in Seawater Simulation Experiments
Source: PLoS One. 2015 Aug 27;10(8):e0136391. doi: 10.1371/journal.pone.0136391 (PMC4552293; doi:10.1371/journal.pone.0136391)
Supplement: S1 Table — Compound 1 (e.g. Desisopropyl Atrazine 1) transitions used for quantitation 174.1/103, other compound designated 2 (Desisopropyl Atrazine 2) is for confirmation, 174.1/68. (DOCX) [file pone.0136391.s001.docx]

S1 Table. Quantification and confirmation ions used for herbicide analysis. Compound 1 (e.g. Desisopropyl Atrazine 1) transitions used for quantitation 174.1/103, other compound designated 2 (Desisopropyl Atrazine 2) is for confirmation, 174.1/68.

| **Precursor** | **Product** |  |  |  |  |  |  |
| --- | --- | --- | --- | --- | --- | --- | --- |
| **Q1** | **Q3** | **Dwell** | **Compound ID** | **DP** | **EP** | **CE** | **CXP** |
| 174.1 | 104 | 30 | Desisopropyl Atrazine 1 | 70 | 10 | 34 | 10 |
| 174.1 | 68 | 30 | Desisopropyl Atrazine 2 | 70 | 10 | 38 | 11 |
| 188.1 | 146 | 30 | Desethyl Atrazine 1 | 65 | 10 | 25 | 10 |
| 188.1 | 104 | 30 | Desethyl Atrazine 2 | 65 | 10 | 38 | 10 |
| 202.1 | 132 | 30 | Simazine 1 | 140 | 10 | 27 | 17 |
| 202.1 | 124 | 30 | Simazine 2 | 140 | 10 | 25 | 17 |
| 253.1 | 171.1 | 30 | Hexazinone 1 | 70 | 10 | 23 | 20 |
| 253.1 | 71 | 30 | Hexazinone 2 | 70 | 10 | 43 | 11 |
| 229.1 | 172.1 | 30 | Tebuthiuron 1 | 65 | 10 | 26 | 11 |
| 229.1 | 116 | 30 | Tebuthiuron 2 | 65 | 10 | 38 | 10 |
| 162 | 127 | 30 | 3,4 Di Cl Analine 1 | 60 | 10 | 30 | 10 |
| 162 | 74 | 30 | 3,4 Di Cl Analine 2 | 60 | 10 | 70 | 10 |
| 216.1 | 174.1 | 30 | Atrazine 1 | 70 | 10 | 25 | 10 |
| 216.1 | 96 | 30 | Atrazine 2 | 70 | 10 | 35 | 11 |
| 221 | 179.1 | 30 | D5 Atrazine 1 | 95 | 10 | 25 | 20 |
| 221 | 69 | 30 | D5 Atrazine 2 | 95 | 10 | 50 | 10 |
| 228.1 | 186 | 30 | Ametryn 1 | 100 | 10 | 25 | 20 |
| 228.1 | 68 | 30 | Ametryn 2 | 100 | 10 | 57 | 11 |
| 235.1 | 72 | 30 | Diuron 1 | 80 | 10 | 26 | 10 |
| 235.1 | 46 | 30 | Diuron 2 | 80 | 10 | 35 | 10 |
| 284.19 | 252 | 30 | Metolachlor 1 | 60 | 10 | 21 | 25 |
| 284.19 | 176 | 30 | Metolachlor 2 | 60 | 10 | 35 | 20 |
| 219 | 161 | 50 | 24D 1 | -70 | -4 | -17 | -10 |
| 221 | 163 | 50 | 24D 2 | -70 | -4 | -17 | -10 |
