# Supplementary material for: Herbicide Persistence in Seawater Simulation Experiments
Source: PLoS One. 2015 Aug 27;10(8):e0136391. doi: 10.1371/journal.pone.0136391 (PMC4552293; doi:10.1371/journal.pone.0136391)
Supplement: S2 Table — (DOCX) [file pone.0136391.s002.docx]

S2 Table. Mean ± SE for each treatment for pH, dissolved oxygen (DO), and total bacterial counts for Experiment 1 and 2.

| Herbicide Treatment | Experiment | Total bacterial counts (x 10^6^) ± SE | pH ± SE | DO (mg l^-1^) ± SE |
| --- | --- | --- | --- | --- |
| Initial seawater used | 1 | 0.94 ± 0.01 | 8.24 ± 0.01 | 6.4 ± 0.03 |
| Control | 1 | 0.94 ± 0.01 | 8.40 ± 0.02 | 6.35 ± 0.05 |
| Herbicide mixture | 1 | 0.91 ± 0.01 | 8.35 ± 0.03 | 6.02 ± 0.01 |
| Herbicide mixture plus mercuric chloride | 1 | 0 | 8.42 ± 0.01 | 6.05 ± 0.08 |
| Initial seawater used | 2 | 2.66 ± 0.4 | 8.20 ± 0.01 | 6.5 ± 0.07 |
| Control D25 | 2 | 2.43 ± 0.01 | 8.21 ± 0.01 | 6.41 ± 0.07 |
| Control L25 | 2 | 2.29 ± 0.02 | 8.22 ± 0.02 | 5.84 ± 0.04 |
| Control D31 | 2 | 2.21 ± 0.01 | 8.24 ± 0.01 | 6.46 ± 0.33 |
| Diuron D25 | 2 | 2.34 ± 0.02 | 8.25 ± 0.01 | 5.85 5 ± 0.15 |
| Diuron L25 | 2 | 2.55 ± 0.02 | 8.24 ± 0.01 | 6.54 ± 0.05 |
| Diuron D31 | 2 | 2.41 ± 0.03 | 8.31 ± 0.01 | 6.30 ± 0.14 |
| Atrazine D25 | 2 | 2.40 ± 0.01 | 8.24 ± 0.01 | 5.52 ± 0.11 |
| Atrazine L25 | 2 | 2.48 ± 0.01 | 8.23 ± 0.01 | 6.22 ± 0.40 |
| Atrazine D31 | 2 | 2.44 ± 0.03 | 8.34 ± 0.01 | 5.30 ± 0.38 |
| Hexazinone D25 | 2 | 2.33 ± 0.26 | 8.24 ± 0.01 | 5.79 ± 0.12 |
| Hexazinone L25 | 2 | 2.85 ± 0.01 | 8.26 ± 0.01 | 5.89 ± 0.06 |
| Hexazinone D31 | 2 | 2.43 ± 0.01 | 8.33 ± 0.01 | 6.14 ± 0.05 |
| Tebuthiuron D25 | 2 | 2.33 ± 0.01 | 8.27 ± 0.01 | 6.15 ± 0.10 |
| Tebuthiuron L25 | 2 | 2.61 ± 0.14 | 8.29 ± 0.04 | 6.55 ± 0.05 |
| Tebuthiuron D31 | 2 | 2.44 ± 0.04 | 8.34 ± 0.01 | 6.40 ± 0.14 |
| Metolachlor D25 | 2 | 2.29 ± 0.02 | 8.26 ± 0.02 | 6.31 ± 0.14 |
| Metolachlor L25 | 2 | 2.64 ± 0.08 | 8.27 ± 0.01 | 6.70 ± 0.12 |
| Metolachlor D31 | 2 | 2.46 ± 0.01 | 8.33 ± 0.01 | 6.30 ± 0.15 |
| 2,4-D D25 | 2 | 2.30 ± 0.01 | 8.24 ± 0.01 | 5.55 ± 0.12 |
| 2,4-D L25 | 2 | 3.01 ± 0.32 | 8.26 ± 0.01 | 6.41 ± 0.19 |
| 2,4-D D31 | 2 | 2.52 ± 0.03 | 8.31 ± 0.02 | 6.19 ± 0.10 |
