# Supplementary material for: Herbicide Persistence in Seawater Simulation Experiments
Source: PLoS One. 2015 Aug 27;10(8):e0136391. doi: 10.1371/journal.pone.0136391 (PMC4552293; doi:10.1371/journal.pone.0136391)
Supplement: S3 Table — NA = not applicable for 0.45 μm filtered seawater. (DOCX) [file pone.0136391.s003.docx]

S3 Table. Physical and chemical information for the 0.45 µm and 20 µm filtered seawater used in experiment 1 and experiment 2 respectively. NA = not applicable for 0.45 µm filtered seawater.

| Parameter | Mean experiment 1 | Mean experiment 2 | Units |
| --- | --- | --- | --- |
| pH | 8.2 | 8.2 |  |
| POC | NA | 0.35 | mg l^-1^ |
| N | NA | 0.05 | mg l^-1^ |
| NPOC/DOC (TOC) | 1.13 | 1.35 | mg l^-1^ |
| DIC | NA | 24.87 | mg l^-1^ |
| NH_4_ | 0.63 | 0.23 | µmol l^-1^ |
| PO_4_ | 0.14 | 0.24 | µmol l^-1^ |
| NO_2_ + NO_3_ | 0.10 | 4.76 | µmol l^-1^ |
| NO_2_ | 0.01 | 0.01 | µmol l^-1^ |
| Si | 8.38 | 8.11 | µmol l^-1^ |
| TDP | 0.31 | 0.31 | µmol l^-1^ |
| TDN | 8.25 | 11.18 | µmol l^-1^ |
| Salinity | 34 | 34 | ‰ |
