# Supplementary material for: Herbicide Persistence in Seawater Simulation Experiments
Source: PLoS One. 2015 Aug 27;10(8):e0136391. doi: 10.1371/journal.pone.0136391 (PMC4552293; doi:10.1371/journal.pone.0136391)
Supplement: S4 Table — Additional results for Experiment 1 for zero order half-life estimates, r2, and the % difference between first order and zero order half-life estimates. (DOCX) [file pone.0136391.s004.docx]

S4 Table. Results of Experiment 1 including first order half-life estimates, slopes, r^2^, average initial concentration, average final concentration, total average degradation. Additional results for Experiment 1 for zero order half-life estimates, r^2^, and the % difference between first order and zero order half-life estimates.

| **Treatment condition** | **Tebuthiuron** | **Ametryn** | **Hexazinone** | | **Simazine** | **Atrazine** | **Diuron** |
| --- | --- | --- | --- | --- | --- | --- | --- |
| *Initial concentration average (x)* | | | | | | | |
| Dark 25°C | 11.00 | 8.26 | | 9.13 | 10.48 | 11.80 | 5.63 |
| Dark 25°C + MC | 10.28 | 8.04 | | 9.04 | 9.72 | 11.62 | 8.99 |
| *Final concentration average (x)* | | | | | | | |
| Dark 25°C | 9.81 | 7.23 | | 8.18 | 9.68 | 10.77 | 5.19 |
| Dark 25°C + MC | 9.55 | 7.71 | | 8.39 | 9.43 | 11.04 | 8.69 |
| *Total degradation (%)* | | | | | | | |
| Dark 25°C | 10.80 | 12.51 | | 10.39 | 7.64 | 8.69 | 7.83 |
| Dark 25°C + MC | 7.14 | 4.19 | | 7.20 | 2.97 | 4.94 | 3.34 |
|  | | | | | | | |
| *Half-life (days), first order with SE* | | | | | | | |
| Dark 25°C | 433 ± 150 | 419 ± 264 | 479 ± 240 | | 579 ± 294 | 631 ± 491 | 513 ± 284 |
| Dark 25°C + MC | 712 ± 160 | 1221 ± 314 | 805 ± 181 | | 2267 ± 751 | 1216 ± 312 | 1743 ± 524 |
| *r^2^ from ln(x), first order* | | | | | | | |
| Dark 25°C | 0.48 | 0.30 | 0.38 | | 0.35 | 0.26 | 0.36 |
| Dark 25°C + MC | 0.14 | 0.09 | 0.14 | | 0.03 | 0.07 | 0.04 |
| *Half-life (days), zero order with SE* | | | | | | | |
| Dark 25°C | 333 ± 42 | 325 ± 53 | | 366 ± 53 | 440 ± 67 | 480 ± 83 | 395 ± 58 |
| Dark 25°C + MC | 537 ± 115 | 894 ± 221 | | 606 ± 133 | 1639 ± 525 | 899 ± 234 | 1318 ± 389 |
| *r^2^ from concentration (x)* | | | | | | | |
| Dark 25°C | 0.48 | 0.30 | | 0.37 | 0.34 | 0.26 | 0.36 |
| Dark 25°C + MC | 0.15 | 0.10 | | 0.14 | 0.03 | 0.08 | 0.05 |
| *Difference (%) between first order half-life and zero order half-life estimate* | | | | | | | |
| Dark 25°C | 23 | 22 | | 24 | 24 | 24 | 23 |
| Dark 25°C + MC | 25 | 27 | | 25 | 28 | 26 | 24 |
