# Supplementary material for: Herbicide Persistence in Seawater Simulation Experiments
Source: PLoS One. 2015 Aug 27;10(8):e0136391. doi: 10.1371/journal.pone.0136391 (PMC4552293; doi:10.1371/journal.pone.0136391)
Supplement: S5 Table — Additional results for Experiment 2 for zero order half-life estimates, r2, and the % difference between first order and zero order half-life estimates. (DOCX) [file pone.0136391.s005.docx]

S5 Table. Results of Experiment 2 including first order half-life estimates, slopes, r^2^, average initial concentration, average final concentration, total average degradation. Additional results for Experiment 2 for zero order half-life estimates, r^2^, and the % difference between first order and zero order half-life estimates.

| **Treatment Condition** | **Atrazine** | | **Diuron** | | **Hexazinone** | **Tebuthiuron** | **24-D** | **Metolachlor** |
| --- | --- | --- | --- | --- | --- | --- | --- | --- |
| *Initial concentration average (x)* | | | | | | | | |
| Dark, 25°C | 10.52 | 8.55 | | 9.68 | | 11.37 | 17.26 | 5.03 |
| Light, 25°C | 10.08 | 8.23 | | 9.24 | | 11.05 | 17.31 | 4.26 |
| Dark, 31°C | 10.12 | 8.62 | | 9.18 | | 11.30 | 17.52 | 3.91 |
| *Final concentration average (x)* | | | | | | | | |
| Dark, 25°C | 9.00 | 7.27 | | 8.81 | | 10.77 | 1.08 | 1.99 |
| Light, 25°C | 8.83 | 5.70 | | 8.42 | | 10.17 | 11.16 | 1.71 |
| Dark, 31°C | 8.87 | 6.38 | | 7.72 | | 10.37 | 0.36 | 1.74 |
| *Total degradation (%)* | | | | | | | | |
| Dark, 25°C | 14.47 | 15.00 | | 8.92 | | 5.26 | 93.74 | 60.37 |
| Light, 25°C | 12.42 | 30.68 | | 8.90 | | 8.02 | 35.54 | 59.95 |
| Dark, 31°C | 12.38 | 25.97 | | 15.91 | | 8.17 | 97.92 | 55.43 |
|  | | | | | | | | |
| *Half-life (days), first order with SE* | | | | | | | | |
| Dark, 25°C | 1606 ± 129 | | 1568 ± 222 | | 2792 ± 172 | 5214 ± 705 | 146 ± 19 | 281 ± 11 |
| Light, 25°C | 2089 ± 338 | | 556 ± 142 | | 2799 ± 467 | 2650 ± 291 | 494 ± 72 | 320 ± 61 |
| Dark, 31°C | 2066 ± 154 | | 818 ± 51 | | 1434 ± 114 | 2840 ± 358 | 88 ± 6 | 298 ± 12 |
| *r^2^ from ln(x), first order* | | | | | | | | |
| Dark, 25°C | 0.88 | 0.70 | | 0.92 | | 0.72 | 0.76 | 0.94 |
| Light, 25°C | 0.65 | 0.51 | | 0.63 | | 0.79 | 0.78 | 0.62 |
| Dark, 31°C | 0.89 | 0.92 | | 0.88 | | 0.75 | 0.94 | 0.97 |
| *Half-life (days), zero order with SE* | | | | | | | | |
| Dark, 25°C | 1257 ± 91 | | 1226 ± 179 | | 2113 ± 92 | 3873 ± 538 | 169 ± 12 | 310 ± 19 |
| Light, 25°C | 1628 ± 270 | | 519 ± 95 | | 2141 ± 304 | 2009 ± 222 | 449 ± 47 | 294 ± 37 |
| Dark, 31°C | 1596 ± 101 | | 686 ± 44 | | 1127 ± 84 | 2139 ± 246 | 157 ± 10 | 307 ± 15 |
| *r^2^ from concentration (x)* | | | | | | | | |
| Dark, 25°C | 0.88 | 0.70 | | 0.92 | | 0.72 | 0.91 | 0.93 |
| Light, 25°C | 0.65 | 0.58 | | 0.64 | | 0.80 | 0.81 | 0.75 |
| Dark, 31°C | 0.89 | 0.92 | | 0.89 | | 0.75 | 0.92 | 0.96 |
| *Difference (%) between first order half-life and zero order half-life estimate* | | | | | | | | |
| Dark, 25°C | 22 | 22 | | 24 | | 26 | -16 | -10 |
| Light, 25°C | 22 | 7 | | 24 | | 24 | 9 | 8 |
| Dark, 31°C | 23 | 16 | | 21 | | 25 | -79 | -3 |
