# Supplementary material for: Herbicide Persistence in Seawater Simulation Experiments
Source: PLoS One. 2015 Aug 27;10(8):e0136391. doi: 10.1371/journal.pone.0136391 (PMC4552293; doi:10.1371/journal.pone.0136391)
Supplement: S6 Table — Repeated measures ANOVA testing significance of degradation over time. (DOCX) [file pone.0136391.s006.docx]

S6 Table. Results of statistical testing of Experiment 1 and Experiment 2. Repeated measures ANOVA testing significance of degradation over time.

| Experiment 1 | DF | F | p |
| --- | --- | --- | --- |
| Diuron | 3,12 | 2.04 | 0.209746 |
| Atrazine | 3,12 | 11.58 | 0.006591 |
| Hexazinone | 3,12 | 12.02 | 0.006002 |
| Tebuthiuron | 3,12 | 34.2 | 0.003610 |
| Ametryn | 3,12 | 48.52 | 0.000134 |
| Simazine | 3,12 | 12.52 | 0.005418 |
| Diuron + MC | 3,12 | 2.55 | 0.151562 |
| Atrazine + MC | 3,12 | 4.01 | 0.069913 |
| Hexazinone + MC | 3,12 | 5.17 | 0.042153* |
| Tebuthiuron + MC | 3,12 | 15.01 | 0.003398* |
| Ametryn + MC | 3,12 | 1.44 | 0.320276 |
| Simazine + MC | 3,12 | 1.96 | 0.221648 |
| Experiment 2 |  |  |  |
| *Dark 25°C* |  |  |  |
| Diuron | 7,24 | 26.44 | 0.000000 |
| Atrazine | 7,24 | 13.3 | 0.000033 |
| Hexazinone | 7,24 | 41.43 | 0.000000 |
| Tebuthiuron | 7,24 | 11.31 | 0.000082 |
| Metolachlor | 7,24 | 76.12 | 0.000000 |
| 2,4-D | 7,24 | 41.99 | 0.000000 |
| *Light 25°C* |  |  |  |
| Diuron | 7,24 | 5.33 | 0.003879 |
| Atrazine | 7,24 | 18.25 | 0.000005 |
| Hexazinone | 7,24 | 16.77 | 0.000008 |
| Tebuthiuron | 7,24 | 13.13 | 0.000035 |
| Metolachlor | 7,24 | 18.88 | 0.000004 |
| 2,4-D | 7,24 | 20.87 | 0.000002 |
| *Dark 31°C* |  |  |  |
| Diuron | 7,24 | 49.97 | 0.000000 |
| Atrazine | 7,24 | 49.4 | 0.000000 |
| Hexazinone | 7,24 | 153.03 | 0.000000 |
| Tebuthiuron | 7,24 | 9.46 | 0.000221 |
| Metolachlor | 7,24 | 125.55 | 0.000000 |
| 2,4-D | 7,24 | 477.19 | 0.000000 |

* Significant different at 28 days but not at day 60
