# Supplementary material for: Herbicide Persistence in Seawater Simulation Experiments
Source: PLoS One. 2015 Aug 27;10(8):e0136391. doi: 10.1371/journal.pone.0136391 (PMC4552293; doi:10.1371/journal.pone.0136391)
Supplement: S7 Table — Two-tailed test for differences between slopes (k). (DOCX) [file pone.0136391.s007.docx]

S7 Table. Results of statistical testing of Experiment 2. Two- tailed test for differences between slopes (k).

| Experiment 2 | DF | F | p |
| --- | --- | --- | --- |
| *Dark 25°C vs Light 25°C* |  |  |  |
| Diuron | 1,44 | 8.99 | 0.0044 |
| Atrazine | 1,44 | 2.52 | 0.1193 |
| Hexazinone | 1,44 | 0.0001 | 0.9893 |
| Tebuthiuron | 1,44 | 14.80 | 0.0004 |
| Metolachlor | 1,44 | 0.39 | 0.5357 |
| 2,4-D | 1,44 | 46.42 | p<0.0001 |
| *Dark 25°C vs Dark 31°C* |  |  |  |
| Diuron | 1,44 | 25.16 | p<0.0001 |
| Atrazine | 1,44 | 5.08 | 0.0292 |
| Hexazinone | 1,44 | 32.87 | p<0.0001 |
| Tebuthiuron | 1,44 | 10.06 | 0.0028 |
| Metolachlor | 1,44 | 0.71 | 0.4028 |
| 2,4-D | 1,44 | 3.17 | 0.0818 |
| *Light 25°C vs Dark 31°C* |  |  |  |
| Diuron | 1,44 | 2.25 | 0.141 |
| Atrazine | 1,44 | 0.004 | 0.9488 |
| Hexazinone | 1,44 | 18.12 | 0.0001 |
| Tebuthiuron | 1,44 | 0.18 | 0.6764 |
| Metolachlor | 1,44 | 0.92 | 0.3426 |
| 2,4-D | 1,44 | 157.54 | p<0.0001 |
