# Supplementary material for: Herbicide Persistence in Seawater Simulation Experiments
Source: PLoS One. 2015 Aug 27;10(8):e0136391. doi: 10.1371/journal.pone.0136391 (PMC4552293; doi:10.1371/journal.pone.0136391)
Supplement: S8 Table — (DOCX) [file pone.0136391.s008.docx]

S8 Table. Relevant literature on the degradation half-life experiments in freshwater.

| **Herbicide** | **Half-life freshwater (days, unless otherwise specified)** | **Water type/conditions specified** | **Reference** |
| --- | --- | --- | --- |
| 2,4-D | ~2 d (suggested) |  | [[1](#_ENREF_1), [2](#_ENREF_2)] |
| 2,4-D | 13 d | pH 7 | [[3](#_ENREF_3)] |
| 2,4-D | Stable | 20°C and pH 7 | [[3](#_ENREF_3)] |
| Ametryn | No value. States: “Slow degradation in UV light” | pH 7 | [[3](#_ENREF_3)] |
| Ametryn | Stable | 20°C and pH 7 | [[3](#_ENREF_3)] |
| Ametryn | 212 d | 5-29°C and pH 7, natural light | [[4](#_ENREF_4)] |
| Atrazine | 335 d | 12-45°C and pH 7, natural light | [[4](#_ENREF_4)] |
| Atrazine | ~2 years (suggested) | Surface | [[1](#_ENREF_1), [2](#_ENREF_2)] |
| Atrazine | 2.6 d | pH 7 | [[3](#_ENREF_3)] |
| Atrazine | 86 d | 20°C and pH 7 | [[3](#_ENREF_3)] |
| Atrazine | Stable, 34–37 d of the experiment | 10°C and 20°C | [[5](#_ENREF_5)] |
| Atrazine | 43 d | 22°C | [[6](#_ENREF_6)] |
| Diuron | ~3 weeks (suggested) |  | [[1](#_ENREF_1), [2](#_ENREF_2)] |
| Diuron | 43 d | pH 7 | [[3](#_ENREF_3)] |
| Diuron | Stable | 20°C and pH 7 | [[3](#_ENREF_3)] |
| Diuron | 33 d |  | [[7](#_ENREF_7), [8](#_ENREF_8)] |
| Hexazinone | Degraded ~20% in 56 d | artificial sunlight | [[4](#_ENREF_4)] |
| Hexazinone | 56 d | pH 7 | [[3](#_ENREF_3)] |
| Hexazinone | 56 d | 20°C and pH 7 | [[3](#_ENREF_3)] |
| Glyphosate | ~2 months (suggested) |  | [[1](#_ENREF_1), [2](#_ENREF_2)] |
| Glyphosate | 69 d | pH 7 | [[3](#_ENREF_3)] |
| Glyphosate | Stable | 20°C and pH 7 | [[3](#_ENREF_3)] |
| Metolachlor | >200 d | pH 1-9 | [[4](#_ENREF_4)] |
| Metolachlor | ~2 months (suggested) | Surface | [[1](#_ENREF_1), [2](#_ENREF_2)] |
| Metolachlor | Stable | pH 7 | [[3](#_ENREF_3)] |
| Metolachlor | Stable | 20°C and pH 7 | [[3](#_ENREF_3)] |
| Simazine | ~3 weeks (suggested) | Surface | [[1](#_ENREF_1), [2](#_ENREF_2)] |
| Simazine | 1.9 d | pH 7 | [[3](#_ENREF_3)] |
| Simazine | 96 d | 20°C and pH 7 | [[3](#_ENREF_3)] |
| Simazine | Stable, 34–37 days of the experiment | River water, 10°C and 20°C | [[5](#_ENREF_5)] |
| Tebuthiuron | Stable | pH 5,7, and 9 at 20°C | [[4](#_ENREF_4)] |
| Tebuthiuron | No value | pH 7 | [[3](#_ENREF_3)] |
| Tebuthiuron | 64 d | 20°C and pH 7 | [[3](#_ENREF_3)] |

**References**

1. Barbash JE. 9.15 - The geochemistry of pesticides. In: Editors-in-Chief:  Holland DHaT, K.K. , editor. Treatise on Geochemistry. Oxford: Pergamon; 2007. p. 1-43.

2. Mackay D, Shiu WY, Ma KC. Illustrated handbook of physical-chemical properties and environmental fate for organic chemicals: Pesticide Chemicals: CRC Press; 1997.

3. PPDB. Pesticide Properties Database University of Hertfordshire. Accessed March 2015

Agriculture & Environment Research Unit (AERU); 2011 [cited March 2015 December 1st]. Available from: <http://sitem.herts.ac.uk/aeru/footprint/index2.htm>.

4. Vencill WK. Herbicide handbook: Weed Science Society of America; 2002.

5. Starner K, Kuivila KM, Jennings B, Moon GE. Degradation rates of six pesticides in water from the Sacramento River, California. US Geological Survey Toxic Substances Hydrology Program Water Resource Investigation Rep. 1999:89-99.

6. Konstantinou IK, Zarkadis AK, Albanis TA. Photodegradation of selected herbicides in various natural waters and soils under environmental conditions. Journal of Environmental Quality. 2001;30(1):121-30.

7. DeLorenzo ME, Fulton MH. Comparative risk assessment of permethrin, chlorothalonil, and diuron to coastal aquatic species. Mar Pollut Bull. 2012;64(7):1291-9. doi: <http://dx.doi.org/10.1016/j.marpolbul.2012.05.011>.

8. USEPA. Reregistration Eligibility Decision for Diuron. Environmental Protection Agency, Washington, DC. 2003:66; Accessed March 2015.
